# Therapeutic anti-glioma effect of the combined action of PCSK inhibitor with the anti-tumoral factors secreted by Poly (I:C)-stimulated macrophages

**DOI:** 10.1038/s41417-020-00286-1

**Published:** 2021-01-05

**Authors:** Mélanie Rose, Marie Duhamel, Soulaimane Aboulouard, Firas Kobeissy, Dominique Tierny, Isabelle Fournier, Franck Rodet, Michel Salzet

**Affiliations:** 1 Université Lille, INSERM, U1192 - Laboratoire Protéomique, Réponse Inflammatoire et Spectrométrie de Masse-PRISM, F-59000 Lille, France; 2 Oncovet Clinical Research (OCR), SIRIC ONCOLille, Villeneuve d’Ascq, France; 3 Department of Psychiatry, McKnight Brain Institute, University of Florida, Gainesville, FL 32611 USA; 4 Institut Universitaire de France, 75000 Paris, France

**Keywords:** Cell biology, CNS cancer, Immunotherapy

## Abstract

Macrophages plasticity is a key feature in cancer progression. Neoplastic cells can alter their immune functions and orient them into a pro-tumoral phenotype. In this context, we developed a new therapeutic strategy to switch macrophages phenotype and reactivate their anti-tumoral functions. We showed a dual activity of a proprotein convertases inhibitor as anti-glioma drug and anti-tumoral macrophages’ reactivation drug. Proprotein convertases are proteases that cleave proteins into functional proteins. Several of their substrates are involved in tumorigenesis and immunosuppression. We combine here proprotein convertases inhibitor with Poly (I:C), a TLR3 ligand, to increase the anti-tumoral activity of macrophages. With mass spectrometry-based proteomics, system biology, combined with biological assays, we established that a stimulation of macrophages with Poly (I:C) increased their secretion of pro-inflammatory cytokines and anti-tumoral factors. 3D invasion assay showed the efficacy of these anti-tumoral factors against mixed glioma cells and macrophages spheroids. Besides, immunofluorescence and proliferation assays showed an additive effect of the proprotein convertases inhibitor and the anti-tumoral factors secreted by Poly (I:C)-treated macrophages on both anti-glioma activity and macrophages anti-tumoral orientation directly in tumor microenvironment, leading to an innovative glioma therapy.

## Introduction

Oncoimmunology is a growing field. Tumors present a complex microenvironment in which several cell types can be found. Among them, immune cells such as macrophages, microglia, T, B, and NK lymphocytes are present [1]. Most of the current strategies target T lymphocytes to reactivate their cytotoxic response. In cancer, T lymphocytes response is inhibited via the recognition of the PD1 receptor located on T lymphocytes and its ligand PDL1 expressed by cancer cells and pro-tumor macrophages [2]. Yet the full activation of these lymphocytes requires the reactivation of pro-tumor macrophages to express a pro-inflammatory and anti-tumor phenotype. Thus, it is necessary to act before this PD1/PDL1 pathway. Cancer cells secrete different anti-inflammatory factors that create an immunosuppressive environment and participate in the recruitment of macrophages. These macrophages are diverted from their anti-tumor function. They participate in tumorigenesis by improving metastatic and angiogenic processes and by inhibiting cytotoxic response [3]. Macrophages must keep their pro-inflammatory phenotype to re-exert their phagocytic activity and to activate the anti-tumor immune response. In this context, proprotein convertases seem to be a clear target. Proprotein convertases (PCs) are proteases of the subtilisin-kexin family that cleave proproteins through limited proteolysis and convert them into bioactive proteins and peptides [4–6]. Mammalian PCs include PCSK1, PCSK2, furin, PCSK4, PCSK5, PCSK6, and PCSK7 which are known to cleave proproteins at paired basic residues [7]. PCs cleave a variety of precursor proteins within the secretory pathway, including neuropeptides, hormones, growth factors, and their respective receptors, adhesion molecule, bacterial toxins, and viral glycoproteins [8]. However, deregulation of these enzymes has been associated with pathological conditions including endocrinopathies [9], Alzheimer’s disease [10], and tumors [11]. Proprotein convertases are implicated in malignancies. PCSKs promote cancer growth and EMT transition [12, 13] by activating tissue-modifying enzymes through at least matrix metalloproteinases or by inhibiting growth factors regulators [4]. The PCSK upregulation is correlated with accelerated tumor progression and poor prognosis. Furin and PACE4 are the most implicated in ovarian [14], prostate cancers [15] and glioma [16]. In this context, inhibiting PCSK/Furin activity has emerged as a therapeutic approach for suppressing cancerous cell-growth and metastatic activity [17, 18]. The specific Furin inhibition using a bifunctional GM-CSF-Furin shRNA construct and an in vitro transduction protocol on the patient’s cancer cells have been efficacious and well-tolerated in phase I-II trials with advanced Ewing’s sarcoma [19] and ovarian cancer patients [20]. Several PCs inhibitors exist such as α1-PDX or inhibitors of PACE4 which reduced tumor progression [18, 21]. In our previous study, we have demonstrated that a peptidomimetic PC inhibitor, that can act against furin and on other PCs such as PC1/3, can reactive macrophages without toxicity and reduce glioma growth [22] and has the advantage to have a broad spectrum on PCs.

However, another way to get anti-tumoral activity has recently emerged, which corresponds to the immune response reactivation.

In this context, it has been demonstrated that Furin and the proprotein convertase 1/3 (PC1/3) are two complementary targets for reinforcing the immune response [23–30]. Furin is critical for the maintenance of peripheral CD4 + Foxp3 + T regulatory cell-dependent immune tolerance and normal CD4 + T helper (Th) cell polarization in vivo [30, 31]. We demonstrated that PC1/3 knock-out mice have high plasma levels of pro-inflammatory cytokines [25]. We also showed that a rat macrophage cell line (NR8383) PC1/3 knockdown (KD) secretes a high concentration of pro-inflammatory chemokines and cytokines [27]. These secreted factors have chemotactic and anti-tumor properties. When stimulated with TLRs ligands, PC1/3 KD macrophages are over-activated and their immune response is exacerbated [26, 27]. Secreted factors by these PC1/3 KD macrophages after TLR4 stimulation with LPS and the anti-tumoral agent Paclitaxel have anti-tumor activities against breast, ovarian, and glioma cancer cells lines. PC1/3 KD macrophages can inhibit invasion of glioma cancer cells (C6 and F98) in 3D spheroid mixed macrophages-glioma culture [26]. We established that PC1/3 KD macrophages secrete high quantities of extracellular vesicles (EVs) [25, 27, 29] which is even more important when TLR4 or TLR9 pathways are triggered in PC1/3 KD macrophages [27, 28]. PC1/3 modulates GRAMD4 levels and thus regulate TLR9 trafficking, to modulate the inflammatory response [29]. Its inhibition reactivates the pro-inflammatory TLR MYD88 NFKB dependent pathway while the anti-inflammatory STAT3 pathway is down-regulated [29].

**Fig. 1:**
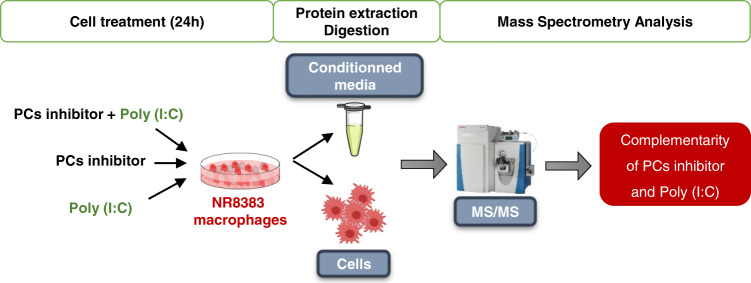
Experimental strategy for proteomic analysis. Graphic summary of the proteomics experiments conducted in this study and the main results.

Taken together, these results point out that PC1/3 KD macrophages can secrete factors that can exert anti-tumoral activity. Thus, we developed a strategy based on PCSK inhibitor to both inactivate the tumor cells and reactivate the tumor associated macrophages (TAM). We established this double effect and the clear ability to reactivate TAM [22]. However, in glioma, the antiviral pathway seems to be more implicated than the TLR4 pathway [32]. We thus investigated in the present paper, the ability to reactivate the anti-tumoral response of macrophages by combining PCSK inhibitor with Poly (I:C) stimulation as a potential anti-glioma therapy. We have demonstrated the secretion by macrophages of antitumoral factors. We have confirmed the combined effect of the treatment by inhibiting tumor growth and reactivating macrophages in mixed spheroids. A graphical abstract describing the proteomic analysis we performed throughout this study is presented in Fig. 1.

## Materials and methods

### Experimental design and statistical rationale

Shotgun proteomics experiments were conducted in biological triplicates (*n* = 3). Spheroid studies were conducted in biological triplicates (*n* = 3) as well as all biological assays. Preactivation spheroid studies and Poly (I:C) treated-NCH82 spheroids were conducted in biological duplicates (*n* = 2) as well as shotgun proteomics experiments on conditioned media-treated NR8383. For the proteomics statistical analysis, extracted proteins or secreted medium proteins presenting as significant by the ANOVA test analysis were used (*p*-value < 0.05). Normalization was achieved using a Z-score with matrix access by rows. For invasion tests, results obtained were depicted through a box plot figure. Significant differences were identified using Tukey’s multiple comparisons test where: *P ≤ 0.05; **P ≤ 0.01; ***P ≤ 0.001; ****P ≤ 0.0001. The tests were made based on an equal variance between the groups. Error bars are represented as s.d.

### Chemicals and reagents

Water (H_2_O), formic acid (FA), acetonitrile (ACN), and trifluoroacetic acid (TFA) were obtained from Biosolve B. V. (Dieuze, France). 2,5 dihydroxybenzoic acid (DHB), ProteoMass MALDI Calibration kit, DL-dithiothreitol (DTT), thiourea, Temozolomide (TMZ), PCs inhibitor (ref. 537076) and iodoacetamide (IAA) were purchased from Sigma-Aldrich (Saint-Quentin Fallavier, France). LysC/Trypsin was purchased from Promega (Charbonnieres, France). Polylysine-coated slides, Dulbecco’s Modified Eagle’s Medium (DMEM), Ham’s F12K, heat-inactivated fetal bovine serum (FBS), trypsin, and phosphate buffer saline (PBS), penicillin and streptomycin were purchased from Thermo-Scientific (Braunschweig, Germany). Poly (I:C) was purchased from Invivogen (San Diego, California, USA). NR8383 is a rat alveolar macrophage cell line (CRL-2192) obtained from ATCC (Manassas, Virginia, USA). The rat C6 glioma cell line was kindly provided by Prof. Dr. Bernd Kaina (Institute of Toxicology, University Medical Center, Mainz, Germany).

### Cell culture

Rat alveolar NR8383 macrophages were cultured in Ham’s F12K medium supplemented with 15% fetal bovine serum and 1% penicillin/streptomycin (100 units per ml). Rat glioma C6 cells and human glioma cell line NCH82 were cultured in DMEM and supplemented with 10% FBS, 1% l-glutamine (2 mM), and 1% penicillin/streptomycin (100 units per ml). All cell lines were cultured at 37 °C in a humidified atmosphere (5% CO_2_).

### Cell supernatant collection and total protein extraction

NR8383 cells were plated on sterile 24 well plates and cultured until they reached confluence. For stimulation, cells were starved overnight in Ham’s F12K medium supplemented with 2% FBS. Cells were then stimulated in serum-free medium with different concentrations of Poly (I:C) (5, 10, or 15 µg/ml). Also, cells were stimulated with 100 µM of PCs inhibitor-associated or not with 10 µg/ml of Poly (I:C) in serum-free medium. At 24 h, cell supernatants were centrifuged at 500 × *g* and passed through a 0.22-µm filter to remove cells and debris. Four hundred microliters of the cell supernatant were collected for each condition. Cells were washed three times with ice-cold PBS and then lysed with RIPA buffer for total protein extraction (150 mM NaCl, 50 mM Tris, 5 mM EGTA, 2 mM EDTA, 100 mM NaF, 10 mM sodium pyrophosphate, 1% NP40, 1 mM PMSF, and 1X protease inhibitors). After 3 × 30 s sonication, cell debris was removed by centrifugation (16,000 × *g*, 10 min, 4 °C), supernatants were collected and protein concentrations were measured using a Bio-Rad Protein Assay Kit, according to the manufacturer’s instructions.

### Filter-aided sample preparation (FASP)

The samples were processed using a shotgun bottom-up proteomic approach. Total protein extract (0.04 mg) was used for FASP analysis as described previously [33–35]. We performed FASP using Microcon devices YM-10 from Millipore (Burlington, Massachusetts, USA) before adding Lys-C trypsin for protein digestion (40 µg/mL in 0.05 M Tris-HCl). An equivalent volume of reduction solution (DTT 0.1 M) was added to each sample followed by an incubation step at 95 °C for 15 min. Then the samples were processed following the filter-aided sample preparation (FASP) protocol using a filter with a nominal molecular weight limit of 10,000 Da (Amicon Ultra-0.5 10 K, Millipore). Briefly, each sample was mixed with 200 µL of denaturant buffer (8 M urea, Tris/HCl 0.1 M, pH 8.5) and transferred to FASP filters. The samples were centrifuged at 14,000 × g, 20 °C, for 15 min. For the alkylation step, 100 µL of 0.05 M of iodoacetamide in denaturant buffer was added to each sample, followed by incubation in the dark for 20 min at room temperature. Samples were washed twice with 100 µL of denaturant buffer followed by two washes with 100 µL of buffer AB (Ammonium bicarbonate 0.05 M). After each washing step, centrifugation was performed at 14,000 × g, 20 °C, for 15 min. The proteins were digested by adding 40 µL of trypsin at 40 µg/ml in buffer AB, and then incubated at 37 °C overnight. The peptides were eluted by adding 50 µL of saline solution (NaCl 0.5 M) and centrifuged at 14,000 × g, 20 °C, for 15 min. The digestion was stopped by adding 10 µL of TFA 5%. The samples were desalted using ZipTip C-18 (Millipore) and eluted with a solution of ACN/0.1% TFA (7:3, v/v). The samples were dried with SpeedVac and resuspended in 20 µL of ACN/0.1% formic acid (0.2:9.8, v/v) just before processing using LC-MS/MS. Experiments were done in biological triplicate (*n* = 3).

### Proteomics analysis of the cell supernatants

Supernatants volume obtained from NR8383 macrophages treated or not with PCs inhibitor or/and Poly (I:C) during 24 h were reduced to 100 µL in a SpeedVac. Cell supernatants were denatured with 2 M urea in 10 mM HEPES, pH 8.0 by sonication on ice. The proteins were reduced with 10 mM DTT for 40 min at 56 °C followed by alkylation with 55 mM iodoacetamide for 40 min in the dark. The iodoacetamide was quenched with 100 mM thiourea. The proteins were digested with 1 µg LysC/Trypsin mixture (Promega) overnight at 37 °C. The digestion was stopped with 0.5% TFA. The samples were desalted using ZipTip C-18 (Millipore) and eluted with a solution of ACN/0.1% TFA (7:3, v/v). The samples were dried with SpeedVac and resuspended in 20 µL of ACN/0.1% formic acid (0.2:9.8, v/v) just before processing using LC-MS/MS. Experiments were done in biological triplicate (*n* = 3).

### LC–MS/MS analysis

Mass spectrometry proteomics analysis of digested proteins was performed using a nano Acquity UPLC system (Waters) coupled with the Q-Exactive Orbitrap mass spectrometer (Thermo Scientific) via a nanoelectrospray source. The samples were separated using online reversed-phase, using a preconcentration column (nanoAcquity Symmetry C18, 5 µm, 180 µm × 20 mm) and an analytical column (nanoAcquity BEH C18, 1.7 µm, 75 µm × 250 mm). The peptides were separated by applying a linear gradient of acetonitrile in 0.1% formic acid (5–35%) for 2 h, at a flow rate of 300 nL/min. The Q- Exactive was operated in data-dependent mode defined to analyze the ten most intense ions of MS analysis (Top 10). The MS analysis was performed with an m/z mass range between 300 to 1600, resolution of 70,000 FWHM, AGC of 3e6 ions and maximum injection time of 120 ms. The MS/MS analysis was performed with an m/z mass range between 200 to 2,000; AGC of 5e4 ion; maximum injection time of 60 ms and resolution set at 17,500 FWHM.

### Protein ID and Data analysis

Proteins were identified by comparing all MS/MS data with the proteome database of the complete reviewed proteome of Rattus norvegicus (Uniprot, release July 2018; 8,054 entries), using the MaxQuant software version 1.6.1.0 [36, 37]. Lys-C trypsin specificity was used for the digestion mode with two missed cleavages. Carbarmidomethylation of cysteines was set as a fixed modification. N-terminal acetylation and methionine oxidation were selected as the variable modifications. For MS spectra, an initial mass tolerance of 6 ppm was selected, and the MS/MS tolerance was set to 20 ppm for HCD data [38]. For identification, the FDR at the peptide spectrum matches (PSMs) and protein level was set to 0.01. Relative, label-free quantification of proteins was performed using the MaxLFQ algorithm integrated into MaxQuant with the default parameters [39]. Analysis of the proteins identified was performed using Perseus software (http://www.perseus-framework.org/) (version 1.6.2.1) [40]. The file containing the information from identification was used with hits to the reverse database, and proteins identified with modified peptides and potential contaminants were removed. Then, the LFQ intensity was logarithmized (log2[x]). Categorical annotation of rows was used to define different groups depending on the concentration of PCs inhibitor used (0, 50, 100, 150 µM). Multiple-samples tests were performed using an ANOVA test with a *p*-value of 0.05 and preserved grouping in randomization. The results were normalized by Z-score and represented as hierarchical clustering. Functional annotation and characterization of identified proteins were obtained using PANTHER software (version 14.0, http://www.pantherdb.org) and STRING (version 10.5, http://string-db.org).

### Sub-network enrichment pathway analysis

Using Elsevier’s Pathway Studio (version 11.0/ /Elsevier), all relationships between the differentially expressed proteins among all conditions were depicted based on the Ariadne ResNet [41]. For proteins identified in the shotgun analysis post-stimulation of cell lines with PCs inhibitor, the Subnetwork Enrichment Analysis (SNEA) algorithm was used to detect the statistically significant altered biological pathways in which the identified proteins are involved. This algorithm uses Fisher’s statistical test to detect any non-random associations between two categorical variables organized by a specific relationship. Also, this algorithm starts by creating a central “seed” from all the relevant identities in the database and builds connections with associated entities based on their relationship with the seed. SNEA compares the sub-network distribution to the background distribution using one-sided Mann–Whitney U-Test and calculates a *p*-value; thus, representing a statistical significance between different distributions. In all analyses that we performed, the GenBank ID was used to form experimental groups based on the different conditions present for analysis. The pathway networks were reconstructed based on biological processes and molecular functions for every single protein, along with its associated targets.

### Biological assays

#### Generation of complete and fractions NR8383 supernatants

NR8383 macrophages were treated with or without 10 µg/mL of Poly (I:C) in complete Ham’s F12K for 24 h. At 24 h, cell supernatants were centrifuged at 200 × g and passed through a 0.22 µm filter to remove cells and debris. Supernatants were then used for spheroids stimulation.

Supernatants were also fractionated with Amicon® Ultra Centrifugal Filters. The different molecular weight cut off were used (100, 50, 30, 10 and 3 kDa) according to the manufacturer’s instruction. The different fractions were used to stimulate C6 cells.

#### Spheroid generation and embedding in a collagen matrix

C6 rat glioma cells associate or not with NR8383 pre-treated or not with 10 µg/mL of Poly (I:C) for 24 h were resuspended in complete Ham’s F12K medium supplemented with 5% of collagen mixture at the final concentration of 8,000 cells of each cell line in 20 µL. For human glioma spheroids generation, we used 8 000 NCH82 glioma cells. The collagen mixture was prepared by mixing 2 ml of PureCol® bovine collagen type I solution (3 mg/mL; Advanced BioMatrix) with 250 µL of 10X minimal essential medium (MEM) (Sigma-Aldrich) and 500 µL of sodium hydroxide 0.1 M. Cells were cultivated in hanging-drop on the lid of a petri dish with PBS during 72 h at 37 °C in a humidified atmosphere (5% CO_2_). The newly formed C6 spheroids and mixed C6/NR8383 spheroids were then implanted in the center of each well of a 24-well plate coated with a collagen mixture described before (one spheroid per well in 400 µL of collagen mixture per well). After cell spheroid embedding, the plate was incubated for 30 min at standard culture conditions to solidify the collagen. Thereafter 400 µL of complete Ham’s F12K medium or conditioned medium from NR8383 was overlaid on the collagen matrix in each well and supplemented with 100 µM of PCs inhibitor. The complete system was incubated for a total of 4 to 6 days. Experiments were done in biological triplicate (*n* = 3). Human spheroids were treated with either 10 µg/mL Poly (I:C) (*n* = 2) alone or with 200 or 300 µM of PCs inhibitor (*n* = 3). The complete system was incubated for a total of 4 days.

#### Quantification of spheroid size and invaded area

After the spheroids were embedded, cell invasion of the spheroid was monitored by digital photography using a Leica DM IL LED Fluo inverted light microscope (Leica DFC450C camera) at room temperature, with the Leica Application Suite (LAS V4.4). Images were acquired every day (day 0 = time of embedding in collagen; pictures were taken immediately after embedding) using a 4x/0.10 objective. Image processing and quantification of spheroids and invasion areas were performed using in-house software. This in-house software takes into account cell density and not the limits of cell migration in the collagen matrix, which is observed independently. The implemented algorithm uses local fluctuations of the image intensity for automated estimation of the invasion magnitude. It is robust enough to handle micrographs of different generation methods and various qualities without the concept of an invasive front of the spheroids [26, 42]. Data of areas are normalized for each day to the relative size of day zero and transformed into the percentage of invasion.

### Apoptosis, necrosis and proliferation assay

C6 cells were seeded into 96-well plates at 30% confluence with conditioned media (CM) from untreated or Poly (I:C)-treated NR8383. These CM were supplemented or not with 100 µM of PCs inhibitor or the same volume of DMSO. The assay was conducted for 72 h. C6 cells were also treated with 600 µM of TMZ as a positive control for apoptosis and 30 µM of Taxol as a positive control for the decrease of proliferation. The Real Time-Glo Annexin V apoptosis and Necrosis reagent (JA1011, Promega) was then added. The luminescence signal corresponding to the exposure of phosphatidylserine on the outer leaflet of cell membranes and was monitored after 72 h using a 96-well plate reader. As well as fluorescence signal corresponding to the release of DNA. Proliferation was measured with the incorporation of BrdU into newly synthesized DNA of actively proliferating cells according to the manufacturer’s instructions (BrdU Cell Proliferation ELISA Kit ab126556, Abcam).

#### Macrophages reactivation quantification by Immunofluorescence and proteomic analysis on C6/NR8383 spheroids and macrophages

Mixed C6/NR8383 spheroids were generated as described before. They were treated with 10 µg/mL of Poly (I:C) or with 100 µM of PCs inhibitor. Spheroids were also treated with CM from non-treated or from Poly (I:C)-treated macrophages supplemented or not with PCs inhibitor for 72 h. For proteomic analysis, same treatment was performed on NR8383 cells. Spheroids and cells were lysed before FASP and LC-MS/MS analysis as described above. For immunofluorescence, mixed spheroids were rinsed with PBS before fixation with 4% paraformaldehyde for 1 h and then washed 3 times in PBS. They were implanted in gelatin 175 mg/mL and frozen at −20 °C and −80 °C. Cryostat (Leica Microsystems, Nanterre, France) was used to cut the spheroids with a thickness of 8 μm per tissue section. The sections where then thaw-mounted on Polylysine-coated slides. Tissue sections were incubated in blocking buffer (0.01% Triton, 1% Normal Donkey Serum (NDS) and 1% BSA in PBS) for 1 h to avoid nonspecific background staining. Mixed spheroids were then incubated overnight at 4 °C with primary antibodies directed against rabbit anti-CD206 (1:1000; from Abcam), goat anti-arginase 1 (1:500; from Novus) and mouse anti-CD68 (1:50; from bio-rad). After 2 washes for 1 min in PBS/Tween 20 0.1% and 3 washes for 5 min in PBS, the mixed spheroids were incubated for 1 h at room temperature with secondary donkey anti-mouse antibody conjugated to Alexa Fluor 555 or with secondary donkey anti-rabbit or donkey anti-goat IgG antibody conjugated to Alexa Fluor 488 diluted in blocking buffer (dilution 1:200; Invitrogen, Carlsbad, CA, USA). Mixed spheroids were rinsed with PBS/Tween 20 0.1% and PBS and counterstained with Hoechst (1:10,000; from euromedex) for 20 min at 4 °C. Finally, after the last PBS washing, spheroids were mounted on a slide with Dako Fluorescent Mounting Medium (Agilent Dako, Santa Clara, CA, USA). Control experiments were performed following the same immunostaining protocol without the primary antibody incubation.

## Data and software availability

Macrophages proteomics data including MaxQuant files and annotated MS/MS have been deposited to the ProteomeXchange Consortium via the PRIDE partner repository with the dataset identifier PXD020258 with a Username: reviewer68417@ebi.ac.uk and Password: mRlwauBi.

## Results

### Poly (I:C) triggers changes in the phenotype and function of macrophages

**Fig. 2:**
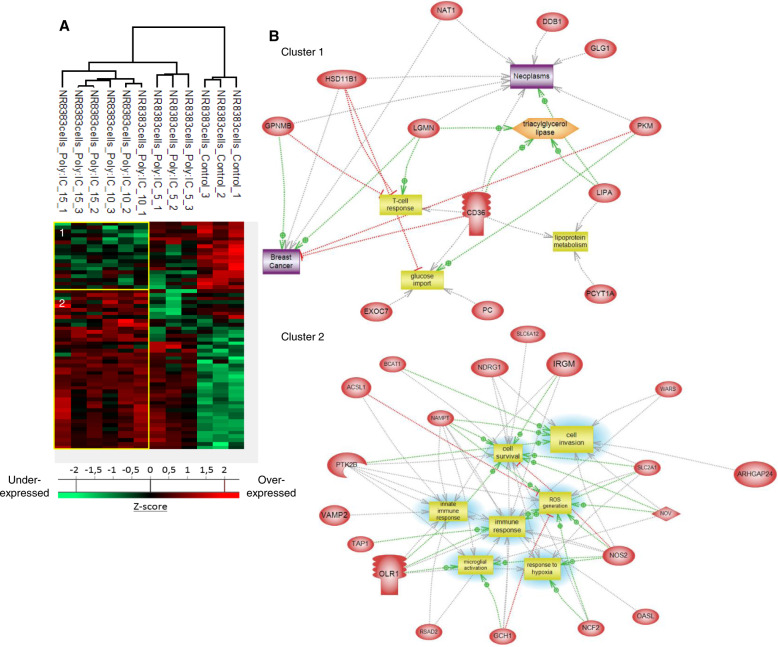
Poly (I:C) induces an immune response by macrophages. **a** NR8383 macrophages were stimulated or not with Poly (I:C) (5, 10 or 15 µg/mL) (*n* = 3). Cells were lysed before FASP and LC–MS/MS analysis. MaxQuant and Perseus software were used for the statistical analysis, and a heatmap was generated to show proteins that were significantly different between treated and untreated macrophages. Two clusters are highlighted. **b** Global pathway analyses of clusters 1 and 2.

A proteomic study was conducted to decipher the molecular impact of different concentrations (5, 10 and 15 µg/mL) of Poly(I:C) on macrophages. Shotgun proteomic analysis of NR8383 macrophages yielded 1632 protein identifications across all the samples. As a criterion of significance, we applied an ANOVA test with a significance threshold of *p* < 0.05. A heatmap was created from which 61 proteins showed a significant difference in LFQ expression between Poly (I:C)-treated NR8383 macrophages and non-treated macrophages (Fig. 2a, Supplementary Data 1). Two branches separate the treated to non-treated Poly (I:C) NR8383 macrophages. Macrophages treated with a concentration of 5 µg/mL Poly (I:C) presents an intermediate profile between non-treated and 10–15 µg/mL Poly (I:C) treated macrophages. Above 10 µg/mL Poly(I:C), the proteome of macrophages is completely different (Fig. 2a). In fact, among the differentially regulated proteins, 18 were downregulated in macrophages treated with 10 and 15 µg/mL of Poly (I:C), including 11β-Hydroxysteroid Dehydrogenase (11β-HSD1), and Transmembrane glycoprotein NMB (Gpnmb) involved in the attenuation of the T cell response (Supplementary Data 1). We also identified, in this cluster of downregulated proteins, Legumain (Lgmn), Lysosomal acid lipase (Lipa) and Platelet glycoprotein 4 (CD36) which are associated with M2 anti-inflammatory polarization of macrophages. Docking protein 3 (DOK3), a proximal negative regulator of TLR signaling, was also found to be downregulated in macrophages after stimulation with 10 and 15 µg/mL of Poly (I:C) (Supplementary Data 1). Systems biology analyses of these downregulated proteins (Fig. 2b, cluster 1) confirm that proteins involved in T Cell response inhibition as well as in breast cancer and neoplasia inhibition are downregulated. In contrast, 43 proteins were upregulated in macrophages after a treatment with 10 and 15 µg/mL of Poly (I:C), including Antigen peptide transporter 1 and 2 (Tap1, Tap2) involved in MHC1 self-antigen presentation, NDRG1 and Interferon-induced GTP-binding protein Mx1 (Mx1), involved in the innate immune response, as well as Interferon-induced guanylate-binding protein 2 (Gbp2) and 2-5-oligoadenylate synthase-like protein 1 (Oasl) (Supplementary Data 1). We also identified, in the upregulated protein cluster, Nicotinamide phosphoribosyltransferase (Nampt) required for T cell-mediated immune responses, Nitric oxide synthase (NOS2) and long-chain acyl-CoA synthetase 1 (ACSL1), markers for the M1 pro-inflammatory phenotype. Ras-related protein Rab-8B (Rab8b) which plays a key role in autophagy processes and Embigin (Emb) involved in cellular adhesion and migration were also found to be upregulated in Poly (I:C)-treated macrophages (Supplementary Data 1), as well as branched-chain-amino-acid aminotransferase (Bcat1) which controls metabolic reprogramming in activated macrophages. Systems biology analyses established that proteins involved in macrophages, innate immunity and thus in immune response activation are more expressed upon Poly(I:C) (Fig. 2b, cluster 2).

### Poly (I:C) triggers the secretion of pro-inflammatory and anti-tumoral factors by macrophages

**Fig. 3:**
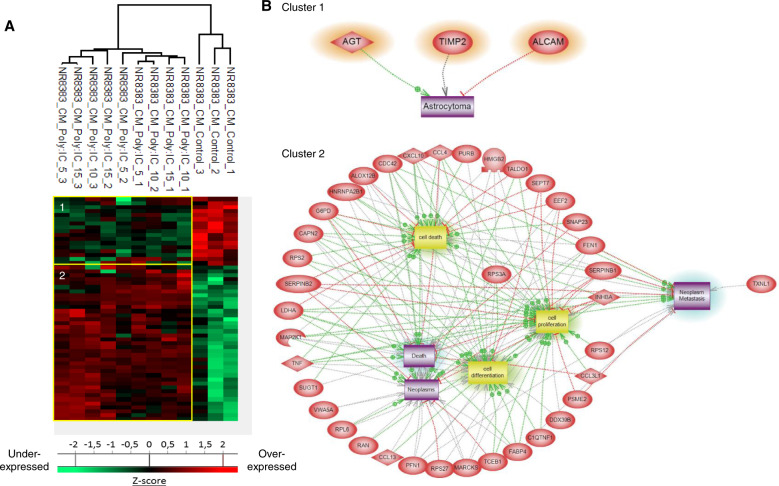
Poly (I:C) induces secretion of pro-inflammatory cytokines by macrophages and immune-related proteins over-expression. **a** NR8383 macrophages were stimulated or not with Poly (I:C) (5, 10 or 15 µg/mL) (*n* = 3). Conditioned media were digested and analyzed with LC–MS/MS. MaxQuant and Perseus software were used for the statistical analysis, and a heat map was generated to show proteins that were significantly different between treated and untreated macrophages. Two clusters are highlighted. **b** Global pathway analyses of clusters 1 and 2.

The same approach was then applied to the conditioned medium of NR8383 treated with Poly (I:C) to analyse the secreted factors (Fig. 3). The proteomic analysis yielded a total of 845 proteins identified across all samples. 56 proteins showed a significant difference in LFQ expression between Poly (I:C)-treated NR8383 macrophages and non-treated macrophages (Supplementary Data 2). 16 proteins were downregulated after stimulation with all the concentration of Poly (I:C) compared to the untreated control (cluster 1, Fig. 3a). Among these, Sulfhydryl oxidase 1 (Qsox1), involved in tumor cell invasion and subsequent metastasis was found downregulated in cluster 1 such as CD166, TIMP2 and AGT knew to be involved in astrocytoma (Fig. 3b, cluster 1). The second cluster regroups 40 proteins which are more secreted by NR8383 macrophages after a stimulation with Poly (I:C), whatever the concentration used (cluster 2, Fig. 3a). Cytokines like CCL2, 3, 4 and 5 or CXCL10, are found in this cluster. These cytokines are important for immune cell recruitment, immune and inflammatory response. Several proteins involved in cell death-like Tumor necrosis factor (TNF), an anti-tumoral factor; Calpain-2 (CAPN2) and Cell division control protein 42 (CDC42) are also more secreted by NR8383 macrophages after a stimulation with Poly (I:C) (cluster 2, Fig. 3b). We also identified as upregulated, Fatty acid-binding protein (Fabp4), involved in the cellular response to the TNF and the inflammatory response, and also High mobility group protein B1 and B2 (Hmgb1 and Hmgb2) which play an important role during V(D)J recombination. Systems biology analyses (Fig. 3b, cluster 2) confirm that all up-regulated proteins found in this cluster can inhibit neoplasia and metastasis. Moreover, Kegg analyses point out that Poly (I:C) triggers proteomic changes both in the cellular component and in the secreted proteins of macrophages. We can observe a secretion of pro-inflammatory and anti-tumoral factors increased in Poly(I:C)-treated macrophages. We thus investigated the anti-tumoral ability of Poly (I:C) combined with PCs inhibitor which has previously demonstrated abilities to inactivate the tumor and activate macrophages [22].

**Fig. 4:**
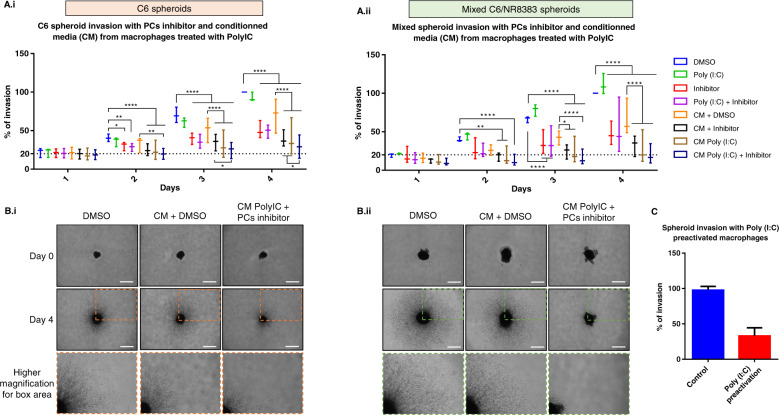
PCs inhibitor associated with the conditioned media of Poly (I:C) activated macrophages has an anti-tumoral effect. C6 (i) and mixed C6/NR8383 (ii) spheroids were incubated with vehicle (DMSO), or with 10 µg/ml of Poly (I:C), or with 100 µM of PCs inhibitor or with both Poly (I:C) and PCs inhibitor. Spheroids were also incubated with conditioned media (CM) from untreated macrophages complemented with PCs inhibitor or vehicle (DMSO) or from macrophages treated with Poly (I:C) for 24 h (i.e., CM Poly (I:C)) complemented with PCs inhibitor or vehicle (DMSO) (*n* = 3). Images of spheroids in the collagen matrix were taken every 24 h for 4 days. **a** Graphic representation showing the percentage of C6 (i) and mixed C6/NR8383 (ii) spheroids invasion. Significant differences were identified using Tukey’s multiple comparisons test with ****p < 0.0001; ***p < 0.001; **p < 0.01. **b** Representative images of the invasion of C6 (i) spheroids and mixed C6/NR8383 (ii) spheroids in the collagen matrix at day 0 and day 4. Spheroids were incubated with (DMSO), or with CM from untreated macrophages complemented with DMSO, or with CM from macrophages treated with Poly (I:C) for 24 h complemented with PCs inhibitor. All images were acquired with an inverted light microscope at 5x magnification. Scale bar: 500 μm. **c** NR8383 was pre-treated or not with 10 µg/mL of Poly (I:C) for 24 h and then used to create mixed spheroids with C6 glioma cells. Graphic representation showing the percentage of invasion at day 3 (*n* = 2).

Thereby, we performed an invasion assay on spheroids made of rat C6 glioma cell line and on mixed spheroids made of both C6 glioma and NR8383 rat macrophages (Fig. 4). The spheroids were cultured for 4 days with Poly (I:C) or with conditioned medium (CM) from Poly (I:C)-treated macrophages combined or not with PCs inhibitor. Their growth and invasion of the matrix by cells migrating out the initial core were monitored over these 4 days. Spheroids containing only C6 cells (Fig. 4a.i) were compared to spheroids containing a mix of C6 and NR8383 cells (Fig. 4a.ii). As we described before, we observed a decrease of spheroid growth and invasion after 2 days of treatment with PCs inhibitor for C6 spheroids and after 3 days of treatment for mixed C6/NR8383 spheroids. No changes in spheroid growth and invasion were observed after a treatment with Poly (I:C) alone compared to the control DMSO. The combination of Poly (I:C) and the PCs inhibitor decreases the invasion of spheroids, but the observed effect is not different from the effect of the inhibitor alone. However, we identified by mass spectrometry some anti-tumoral factors in the conditioned medium of Poly (I:C)-treated macrophages (Fig. 3). Thus, to investigate the anti-tumoral activity of these factors we treated both types of spheroids with conditioned medium from macrophages previously treated or not with Poly (I:C). These conditioned media were associated or not with PCs inhibitor to assess the complementarity of both agents. C6 spheroids and mixed C6/NR8383 spheroids growth and invasion were decreased after 2 days of treatment with conditioned medium from Poly (I:C)-treated macrophages (Fig. 4b.i, 4b.ii). This effect increased progressively across time. Compared to the conditioned medium from untreated-macrophages, a statistical difference of more than 30% was observed after 4 days of treatment. No additive effect was observed when PCs inhibitor was combined with conditioned medium from Poly (I:C)-treated macrophages because of the low invasion rate of spheroids. Taken together, invasion assays and proteomic demonstrated secretion of anti-tumoral factors by macrophages after Poly (I:C) treatment.

Taken together, these results showed that Poly(I:C) treatment alone is not sufficient to trigger the anti-tumor activity of macrophages co-cultured with glioma cells whereas the conditioned medium of Poly(I:C) treated macrophages can inhibit glioma cells invasion. Thus, we next investigated the effect of macrophages Poly(I:C) pre-activation before their co-culture with glioma cells. As shown on Fig. 4c, pre-activated macrophages can keep their anti-tumoral activity and inhibit glioma cells invasion up to 50% (Fig. 4c).

**Fig. 5:**
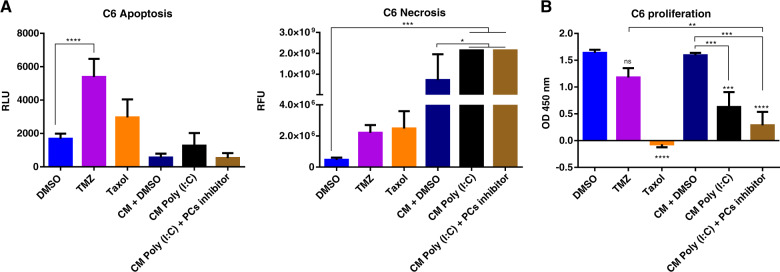
Conditioned media from Poly (I:C) treated-macrophages leads to the C6 glioma cells necrosis. C6 cell line was incubated with Poly (I:C) (10 µg/ml), PCs inhibitor (100 or 300 µM), TMZ (600 µM), the conditioned media (CM) from untreated macrophages complemented with DMSO, the CM from Poly (I:C) pretreated macrophages complemented with DMSO, PCs Inhibitor (100 µM) or with Taxol (30 µM). **a** Necrosis was monitored with RealTime-Glo™ Apoptosis and Necrosis Assay. **b** The C6 proliferation was studied with BrDU incorporation. All the results were representative of three independent experiments. Significant differences were identified using Tukey’s multiple comparisons test with ****p < 0.0001; ***p < 0.001; **p < 0.01 *p < 0.05; *ns* = non significate.

We then investigated the impact of anti-tumoral factors secreted by macrophages on the C6 death and proliferation (Fig. 5). We measured the exposure of phosphatidylserine (PS) on the outer leaflet of cell membranes during the apoptotic process and used a DNA-binding dye to differentiate apoptosis from necrosis after 72 h of treatment (Fig. 5a). C6 glioma cells were treated with temozolomide (TMZ) as a positive control for apoptosis. Compared to the non-treated cells and positive control, no signal for exposure of PS in C6 cells was recorded after the different treatments. However, a very high fluorescence signal due to the DNA dye was observed after a treatment with the conditioned medium from Poly (I:C) treated-macrophages, associated or not with the PCs inhibitor. This signal reflects a loss of membrane integrity and therefore cell death by necrosis. Moreover, a BrdU test correlates these results (Fig. 5b). Indeed, the proliferation of cells is decreased when the cells are treated with the conditioned medium from macrophages treated with Poly (I:C). This effect was more significant when the PCs inhibitor was associated with the conditioned medium from macrophages treated with Poly (I:C).

Finally, to know the approximative molecular weight of the anti-tumoral factors, we performed a viability assay of C6 cancer cells in presence of the different molecular weight fractions of the conditioned medium coming from Poly (I:C) stimulated or unstimulated macrophages. We identified that the fractions containing the anti-tumoral activity are the ones with a molecular weight ranging between 50–100 kDa (Supplementary Fig. 1).

### Poly (I:C) and PCs inhibitor are complementary for macrophages reactivation

**Fig. 6:**
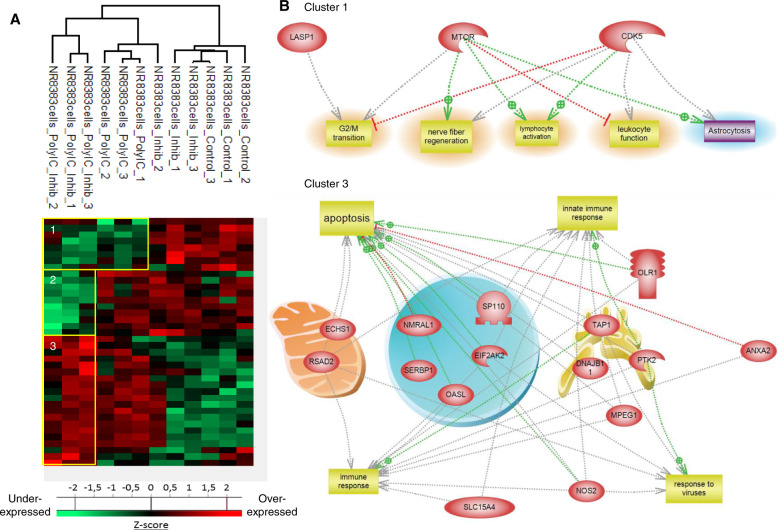
Poly (I:C) associated with PCs inhibitor induces immune-related proteins over-expression and underexpression of pro-tumoral proteins. **a** NR8383 macrophages were stimulated with 10 µg/mL of Poly (I:C) associated or not with 100 µM of PCs inhibitor (*n* = 3). Cells were lysed before FASP and LC–MS/MS analysis. MaxQuant and Perseus software were used for the statistical analysis, and a heat map was generated to show proteins that were significantly different between treated and untreated macrophages. Three clusters are highlighted (1, 2 and 3). **b** Global pathway analyses of clusters 1 and 3.

**Fig. 7:**
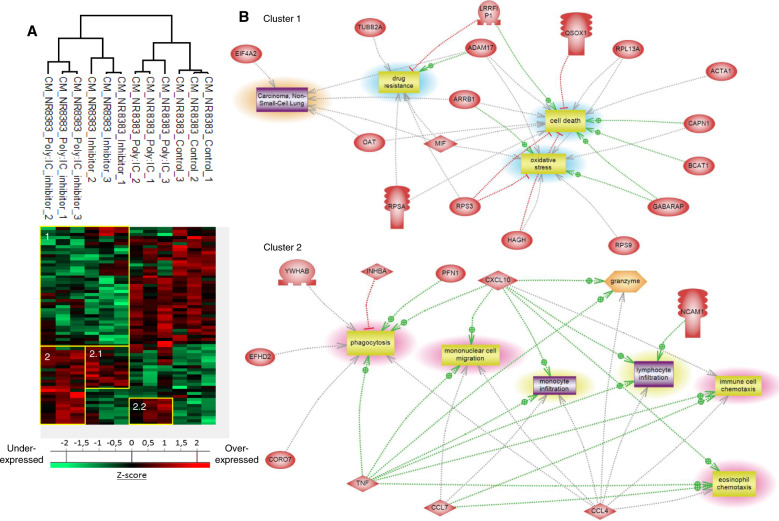
Poly (I:C) associated with PCs inhibitor induces secretion of pro-inflammatory cytokines by macrophages. NR8383 macrophages were stimulated with 10 µg/mL of Poly (I:C) associated or not with 100 µM of PCs inhibitor (*n* = 3). Conditioned media were digested and analyzed with LC–MS/MS. **a** MaxQuant and Perseus software were used for the statistical analysis, and a heatmap was generated to show proteins that were significantly different between treated and untreated macrophages. Two clusters are highlighted (1 and 2). **b** Global pathway analyses of clusters 1 and 2.

A proteomic study was conducted to decipher the molecular impact of the association between Poly (I:C) and PCs inhibitor on macrophages and their secretion (Figs. 6 and 7). First, shotgun proteomic analysis of NR8383 cells yielded 1,717 protein identification across all samples. As a criterion of significance, we applied an ANOVA test with a significance threshold of *p* < 0.05. A heatmap was created from which 38 proteins showed a significant difference in LFQ expression between treated and non-treated NR8383 macrophages (Fig. 6a, Supp Data 3). Two branches separate the Poly (I:C) +/− PCs inhibitor-treated macrophages to untreated and PCs inhibitor-treated macrophages. Among these proteins, 7 were downregulated in macrophages treated with Poly (I:C), associated or not with PCs inhibitor (Fig. 6a, cluster 1). In this protein cluster, we found the transmembrane glycoprotein NMB (Gpnmb), and the Microphthalmia-associated transcription factor (Mitf), a transcription factor that regulates the expression of genes with essential roles in cell differentiation, proliferation and survival. 10 proteins are downregulated only after a treatment with Poly (I:C) associated with PCs inhibitor (Fig. 6a, cluster 2). Among these proteins, we found 2,5-phosphodiesterase 12 (Pde12) a negative regulator for antiviral and antitumor functions induced by interferons and Eukaryotic translation initiation factor 3 subunit E (Eif3e) which is essential for proliferation and survival of glioblastoma cells. In a third cluster, we found upregulated proteins after a treatment with Poly (I:C), associated or not with PCs inhibitor. In this cluster, we find several proteins previously described as upregulated after a Poly (I:C) treatment (Fig. 2), as Gbp2, Tap1, Mx1 and Oasl involved in innate immune response and Nos2 marker for the M1 pro-inflammatory phenotype. We also found UDP-glucose 6-dehydrogenase (Ugdh), a component of the extracellular matrix and F-actin-capping protein subunit alpha-1 (Capza1) which regulate actin cytoskeleton remodeling.

The same approach was then applied to the conditioned medium of NR8383 treated with Poly (I:C) associated or not with PCs inhibitor to analyse the secreted factors (Fig. 7, Supplementary Data 4). The proteomic analysis yielded a total of 1152 proteins identified across all samples. 66 proteins showed a significant difference in LFQ expression between treated NR8383 macrophages and non-treated macrophages. Two branches separate the PCs inhibitor + /− Poly (I:C) treated macrophages to untreated and Poly (I:C) treated macrophages. Compared to the untreated control, 39 proteins were downregulated after stimulation with PCs inhibitor, associated or not with Poly (I:C). Conditioned medium from cells treated with only Poly (I:C) show the same expression profile than the untreated cells (Fig. 7a). The down-secretion by macrophages of migration-related protein macrophage migration inhibitory factor (MIF), also known to be involved in glioblastoma progression, was previously described after treatment with PCs inhibitor [22]. In this cluster, we also found Qsox1, as described in Fig. 3a. Several proteins involved in different tumorigenesis processes are down-secreted by macrophages after treatment with PCs inhibitor. Among these proteins, we identified Disintegrin and metalloproteinase domain-containing protein 17 (Adam17), known to promotes glioma cell malignant phenotype and Branched-chain-amino-acid aminotransferase (Bcat1) which promotes cell proliferation in glioma. Calcium/calmodulin-dependent protein kinase type II subunit delta (Camk2d) plays a critical role in the invasion and metastasis properties of glioma cells and is down-secreted by macrophages after treatment with PCs inhibitor. Insulin-like growth factor II (Igf2) which plays a role in reprogramming macrophage into the anti-inflammatory phenotype was also down-secreted by macrophages after PCs inhibitor treatment. A second protein cluster can be highlighted in which we found 25 proteins over-expressed after a treatment with PCs inhibitor associated with Poly (I:C) (cluster 2, Fig. 7a). 12 of these are also over-expressed after treatment with PCs inhibitor but not after a treatment with Poly (I:C) (cluster 2.1, Fig. 7a). Among these proteins, we found Ubiquitin carboxyl-terminal hydrolase 15 (Usp15) which positively regulates type I interferon responses and thereby antiviral immune response. The other 13 proteins of the second cluster are over-expressed after a treatment with Poly (I:C) and after treatment with both Poly (I:C) and PCs inhibitor (cluster 2.2, Fig. 7a). Among these proteins, we found Cxcl10, Ccl3, Ccl4 and Tnf several cytokines described above (Fig. 3a). We also found, as described above (Fig. 3a) Gbp2 and Hnrnpa2b1, which initiate and amplify the innate immune response, and Inhba M1 pro-inflammatory phenotype marker.

**Fig. 8:**
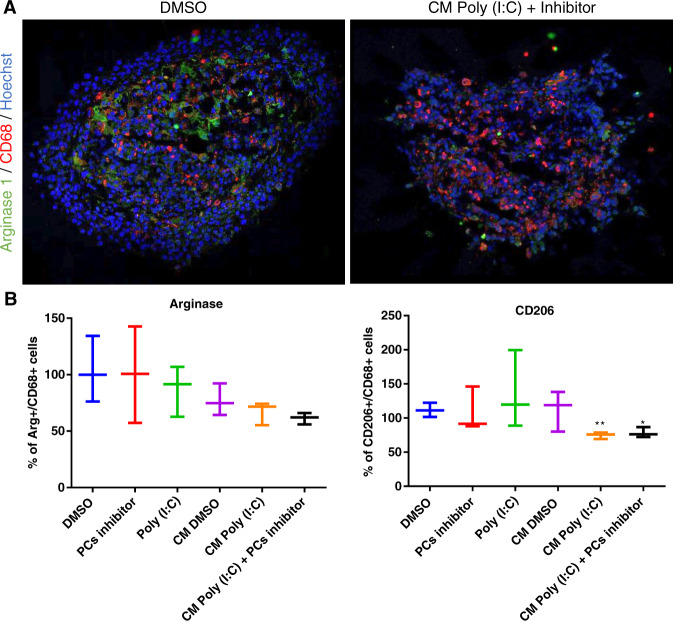
Conditioned media from Poly (I:C) treated macrophages combine with PCs inhibitor decrease the Arginase expression into mixed spheroids. Mixed C6/NR8383 spheroids were incubated for 24 h with vehicle (DMSO), or with 10 µg/mL of Poly (I:C) or with 100 µM of PCs inhibitor (*n* = 3). Spheroids were also incubated with conditioned media (CM) from untreated macrophages complemented with vehicle (DMSO) or from macrophages treated with Poly (I:C) for 24 h (i.e., CM Poly (I:C)) complemented with PCs inhibitor. **a** Confocal images of mixed spheroids after cryosection and immunofluorescence staining for Hoechst (blue), CD68 (red) and Arginase 1 (green) are shown. **b** Percentage of macrophages (CD68) expressing arginase 1 or CD206. Significant differences were identified using T test with **p < 0.01 and *p < 0.05.

Taken together, these results show a complementarity between Poly (I:C) and PCs inhibitor in the macrophages reactivation. The Poly (I:C) triggers more proteins changes in the cell component than the PCs inhibitor and, on the contrary, PCs inhibitor triggers more changes in the secretion profile of macrophages. Besides, several proteins show expression changes only when macrophages are stimulated with both Poly (I:C) and PCs inhibitor (cluster 2, Fig. 6a and cluster 2, Fig. 7a). Moreover, we showed with immunofluorescence on mixed macrophages-cancer cells spheroids a decrease in the expression of Arginase 1 and CD206, both markers of the pro-tumoral phenotype, when they were cultivated in presence of the conditioned medium from Poly (I:C)-treated macrophages. The numbers of CD206+ and arginase+ macrophages seem to be lower when PCs inhibitor was combined with conditioned medium from Poly (I:C) treated macrophages (Fig. 8). To confirm these results, we analyzed the expression of pro-inflammatory and anti-inflammatory markers in the proteomic data (Supplementary Fig. 2). A decrease of anti-inflammatory markers expression is measured for CD206, STAT3 and Arginase 1 in macrophages treated with the conditioned medium from Poly (I:C)-treated macrophages with and without the addition of the PCs inhibitor. We also observed a higher expression of the pro-inflammatory marker MHC I in these macrophages even when they are associated with glioma cells in spheroids. We finally confirmed on spheroids made of NCH82 human glioma cells that the Poly (I:C) and PCs inhibitor are highly efficient on tumor invasion decrease 4 days after treatment (Supplementary Fig. 3).

## Discussion

In the present study, we have demonstrated, based on shot-gun proteomic studies, that Poly (I:C) at a concentration ranging between 10 and 15 µg/mL of Poly (I:C) changes the phenotype of macrophages. Their immune functions seem to be activated and their phenotype is close to a pro-inflammatory phenotype. A lower concentration (5 µg/mL) is not enough to observe this activation state. This pro-inflammatory phenotype is based on an increase of expression of proteins involved in self-antigen presentation (Tap1, Tap2), the implication in anti-viral response through interferon intracellular signaling activation, Nitric oxide synthase (NOS2) and long-chain acyl-CoA synthetase 1 (ACSL1) level increase, as well as the involvement of factors such like Embigin known being implicated in cellular adhesion and migration or the branched-chain-amino-acid aminotransferase involved in metabolic reprogramming in activated macrophages. Similarly, analyses of the secreted factors revealed the presence of pro-inflammatory cytokines (CCL2, CCL3, CCL4 and CCL5 or CXCL10) and TNF. These results are in line with the ones obtained on rodent or human poly(I:C)-stimulated macrophage showing the production of TNF and or CXCL-10 (IP-10) [43, 44]. CXCL10 is known to play a significant role in leukocyte homing to inflamed tissues, increased production of CXCL10 may exacerbate inflammation [43]. Thus, augmented CXCL10 production by macrophages, may further exacerbate the inflammatory response and increase T cell recruitment which is expected in oncoimmune therapy. Based on these results, we tested the effects of such activated medium issued from Poly(I:C) treated macrophages on glioma cells in 3D culture. Compared to the conditioned medium from untreated-macrophages, a statistical difference of invasion of glioma cells of more than 30% was observed after 4 days of treatment. Thus, invasion assays and proteomic demonstrated secretion of anti-tumoral factors by macrophages after Poly (I:C) treatment. We confirmed the relevance of the conditioned medium from Poly (I:C) treated-macrophages with several biological essays. We established that glioma proliferation is decreased when the glioma cells are treated with the conditioned medium from macrophages treated with Poly (I:C). These results are in the same line as the ones of Maeda and collaborators [45]. This team has demonstrated that macrophages differentiated in the presence of pancreatic tumor cells (PANC1 and PT45) and treated with Poly (I:C) secrete more CXCL10 and CCL5 which trigger the cytotoxic activity of TC-Mϕ against cancer cells [45]. We have also demonstrated the efficacy of Poly (I:C) and PCs inhibitor on the invasion decrease of spheroids made with human glioma cell line NCH82. In our work, we identified that the fractions containing the anti-tumoral activity in the conditioned medium correspond to fractions with a molecular weight ranging between 50–100 kDa. We finally established using immunofluorescence studies with anti-CD68, anti-arginase and anti-CD206 that the combined treatment leads to the activation of TAMs with an M1 phenotype directly into the tumor spheroids whereas TAMs express an M2-like phenotype without treatment.

Altogether, we established that the combination of Poly (I:C) macrophages conditioned medium and the PCs inhibitor allowed the reactivation of tumor-associated macrophages which consequently inhibits tumor proliferation and invasion. The next step we will be to hunt in Poly (I:C) macrophages conditioned medium the anti-tumoral factors. These factors are secreted and could be contained in extracellular vesicles (EVs) as we previously showed with microglia cells [16, 46]. In this context, an innovative therapeutic strategy based on EVs from Poly (I:C)-stimulated macrophages incorporating PCs inhibitor will be envisaged and needs further experiments.

## Supplementary information

Supp Material

Supp.figure

Supp. data 1

Supp. Data 2

Supp. data 3

Supp. data 4
