# Supplementary material for: Therapeutic anti-glioma effect of the combined action of PCSK inhibitor with the anti-tumoral factors secreted by Poly (I:C)-stimulated macrophages
Source: Cancer Gene Ther. 2021 Jan 5;29(1):22–36. doi: 10.1038/s41417-020-00286-1 (PMC8761570; doi:10.1038/s41417-020-00286-1)
Supplement: Supplementary file 1 — Supp Material [file 41417_2020_286_MOESM1_ESM.docx]

**Supp. Figure 1: Anti-tumoral factors secreted by Poly (I:C) pre-treated macrophages have a molecular weight greater than 100 kDa.** Conditioned media from Poly (I:C) pre-treated macrophages and untreated macrophages were split in different fraction with molecular weight filter. C6 rat glioma cells were incubated with complete conditioned media from Poly (I:C) pre-treated macrophages or with different fractions of this conditioned media. The cell density of C6 cells was determined by MTS assay. The assays were conducted for 3 days. All the results were representative of three independent experiments. Significant differences were identified using Tukey's multiple comparisons test with ** p<0,01.

**Supp. Figure 2: Conditioned media from Poly (I:C) treated macrophages combine with PCs inhibitor orient macrophages towards pro-inflammatory phenotype.** NR8383 (NR) macrophages and mixed spheroids were stimulated with conditioned media (CM) from untreated macrophages complemented with vehicle (DMSO) or PCs inhibitor or with CM from macrophages treated with Poly (I:C) for 24h (i.e CM Poly (I:C)) complemented with DMSO or PCs inhibitor. Cells and spheroids were lysed before FASP and LC-MS/MS analysis. MaxQuant and Perseus software were used for the protein identification. Graphs represent LFQ intensities showing expression of CD206 (MRC1), STAT3, Arginase 1 and MHC I.

**Supp. Figure 3: PCs inhibitor and Poly (I:C) has an anti-tumoral effect on the human glioma cell line NCH82.** NCH82 spheroids were incubated with vehicle (DMSO), or with 10µg/ml of Poly (I:C), or with 200µM or 300µM of PCs inhibitor. Images of spheroids in the collagen matrix were taken every 24h for 4 days. A) Graphic representation showing the percentage of spheroids invasion after a treatment with Poly (I:C) (n=2) or PCs inhibitor (n=3). Significant differences were identified using Tukey's multiple comparisons test with **** p<0.0001 ; ** p<0,01 and * p<0.05. B) Representative images of the invasion of untreated and Poly (I:C) treated NCH82 spheroids in the collagen matrix at day 0 and day 4. All images were acquired with an inverted light microscope at 5x magnification. Scale bar: 100 μm.

**Supp. Data 1**: List of identified protein from cluster 1 & 2 of NR8383 cells treated with Poly (I:C).

**Supp. Data 2**: List of identified protein from cluster 1 & 2 of conditioned media from NR8383 treated with Poly (I:C).

**Supp. Data 3**: List of identified protein from cluster 1, 2 & 3 of NR8383 cells treated with Poly (I:C) associated or not with PCs inhibitor.

**Supp. Data 4**: List of identified protein from cluster 1 & 2 of conditioned media from NR8383 treated with Poly (I:C) associated or not with PCs inhibitor.
